# A multi-modal deep learning solution for precise pneumonia diagnosis: the PneumoFusion-Net model

**DOI:** 10.3389/fphys.2025.1512835

**Published:** 2025-03-12

**Authors:** Yujie Wang, Can Liu, Yinghan Fan, Chenyue Niu, Wanyun Huang, Yixuan Pan, Jingze Li, Yilin Wang, Jun Li

**Affiliations:** ^1^ College of Information Engineering, Sichuan Agricultural University, Ya’an, China; ^2^ Deep Vision Agriculture Lab, Sichuan Agricultural University, Ya’an, China; ^3^ College of Science, Sichuan Agricultural University, Ya’an, China; ^4^ Agricultural Information Engineering Higher Institution Key Laboratory of Sichuan Province, Ya’an, China; ^5^ Ya’an Digital Agricultural Engineering Technology Research Center, Ya’an, China

**Keywords:** pneumonia classification, deep learning, multimodal framework, clinical data integration, PneumoFuison-Net

## Abstract

**Background:**

Pneumonia is considered one of the most important causes of morbidity and mortality in the world. Bacterial and viral pneumonia share many similar clinical features, thus making diagnosis a challenging task. Traditional diagnostic method developments mainly rely on radiological imaging and require a certain degree of consulting clinical experience, which can be inefficient and inconsistent. Deep learning for the classification of pneumonia in multiple modalities, especially integrating multiple data, has not been well explored.

**Methods:**

The study introduce the PneumoFusion-Net, a deep learning-based multimodal framework that incorporates CT images, clinical text, numerical lab test results, and radiology reports for improved diagnosis. In the experiments, a dataset of 10,095 pneumonia CT images was used-including associated clinical data-most of which was used for training and validation while keeping part of it for validation on a held-out test set. Five-fold cross-validation was considered in order to evaluate this model, calculating different metrics including accuracy and F1-Score.

**Results:**

PneumoFusion-Net, which achieved 98.96% classification accuracy with a 98% F1-score on the held-out test set, is highly effective in distinguishing bacterial from viral types of pneumonia. This has been highly beneficial for diagnosis, reducing misdiagnosis and further improving homogeneity across various data sets from multiple patients.

**Conclusion:**

PneumoFusion-Net offers an effective and efficient approach to pneumonia classification by integrating diverse data sources, resulting in high diagnostic accuracy. Its potential for clinical integration could significantly reduce the burden of pneumonia diagnosis by providing radiologists and clinicians with a robust, automated diagnostic tool.

## 1 Introduction

Pneumonia is a very heterogeneous respiratory disease that every year affects millions worldwide, leading to serious complications and death among the many cases of children, seniors, and immunocompromised patients (Torres et al., 2021). Traditional diagnosis of pneumonia mainly relies on clinical symptoms, physical examination, and image studies, such as X-rays and computing tomography or CT scans (Franquet, 2001). However, in the presence of different pneumonia forms, diagnostic methods are often faced with a lack of sufficient sensitivity and specificity. The significant overlapping symptomatic manifestations of both viral and bacterial pneumonia often pose difficulties for the clinicians in making the right diagnoses based on one source of information only (Cawcutt and Kalil, 2017). Accordingly, medically, there is still very significant importance for improving diagnostic accuracy and efficiency in some complex cases (Castaneda et al., 2015).

Multimodal data fusion has been considered during the last few years as a promising way of enhancing diagnostic precision in complex diseases through integrated analysis by exploiting heterogeneous data from multiple sources, such as CT images, case histories, laboratory test results, and radiologic reports (Chen et al., 2019). It comes intrinsically from information gain theory in information science-that with the introduction of more independent data sources, models can gain more relevant information than single-modal data, thereby reducing diagnostic uncertainty (Crupi et al., 2018). Moreover, research in psychology and cognitive science shows that human physicians also make most of their complicated decisions by integrating several information sources, such as imaging data, laboratory test indicators, and clinical presentation (Mu et al., 2020). Therefore, the fusion of multimodal data not only follows the information integration theory in the deep learning field but also simulates the decision-making process of clinical physicians.

In the medical context, different modal data bear rich and complementary information (Bramon et al., 2012). The imaging data reflect intuitive anatomical and pathological features, whereas the laboratory test results reflect physiological and pathological states of the organism (Tian et al., 2021). For example, though viral and bacterial pneumonia present similar imaging features of pulmonary shadows, the WBC count is usually normal, and the lymphocyte percentage is elevated in the laboratory test in the viral pneumonia patients, while notably elevated CRP and WBC count could always be seen in bacterial pneumonia (Titova et al., 2018). Existing multimodal approaches, however, often adopt simplistic fusion strategies (e.g., early concatenation or late averaging), which fail to model dynamic interactions between modalities. PneumoFusion-Net addresses this gap through its Swin Transformer-based dynamic attention mechanism, enabling adaptive weighting of CT, text, and numerical data based on diagnostic relevance.This is because the complementarity of the sources is what actually enables multimodal fusion to effectively enhance diagnostic accuracy in difficult clinical scenarios (Suk et al., 2014).

With advanced computational capability and deep learning algorithms, how to effectively integrate data from different modalities has become a kind of frontier research topic (Lundervold and Lundervold, 2018). Traditional diagnostic models often rely on single-modality diagnosis-for instance, analyzing CT images with CNNs. However, these methods frequently cannot handle multidimensional feature presentations of complex pathologies. These models, on the other hand, possess the capability for multi-modal deep learning: to process data of different natures by various neural network modules and perform feature fusion at either intermediate layers or decision layers. This is based on the theoretical assumption that deep learning models can abstract high-dimensional feature representations of data through multi-layer neural networks and jointly model these features through certain fusion mechanisms, such as attention mechanisms or Transformer architecture, hence capturing the correlations between different modalities (Choi and Lee, 2023).

Recently, the attention mechanism-based model family, including Transformers has shown excellent performance in the field of fusing multimodal data (Xu et al., 2022). The Transformer model with a self-attention mechanism is competent in modeling long-distance dependencies between different modal data and capturing global correlations among them. It can be fit for medical data fusion since data from different modalities, such as CT images and clinical texts, are often intertwined in various ways both in space and time (Moghadam et al., 2022). It can be used to compare, for example, the progress of a patient’s disease as represented by laboratory tests and CT scans taken at different times, in order to dynamically weight different modal data for the purpose of generating more accurate diagnostic results using the Transformer architecture (Pu et al., 2020).PneumoFusion-Net extends this capability by integrating Swin Transformer’s shifted window mechanism, which reduces computational complexity while maintaining global interaction across modalities—a critical advantage over conventional Transformers in processing high-resolution CT data.

While deep learning of multimodal has demonstrated enormous potential and found great application in research domains, there are still manifold challenges associated with its clinical applications (Huang et al., 2020). First, there is a large difference in data format, dimensionality, scale, and acquisition methods across the different modalities. For example, highly structured three-dimensional data are represented in the CT image data, while often unstructured free-text data is available for clinical texts (Seinen et al., 2022). How to effectively extract and fuse features while ensuring integrity of the data is a big challenge. The heterogeneity of medical data manifests not only in the formats of data but also in the quality and annotation standard of the data. Due to the challenges above, early fusion, late fusion, and intermediate fusion have been proposed in a series of fusion strategies. Techniques based on self-supervised learning and transfer learning were also introduced to enhance model generalization over multi-center and heterogeneous datasets (Stahlschmidt et al., 2022).

The model interpretability problem is an essential preventing factor for it to be used clinically (Elshawi et al., 2019). During clinical decision-making, the physicians are interested not only in the diagnostic outcome developed by AI models but also in knowing what features the model relied on to make the judgment (Neves and Marsh, 2019). So, lots of interpretability methods have been proposed recently, including Grad-CAM and attention mechanism-based explanation frameworks (Zhang et al., 2021). These techniques give doctors not only an insight into the model decision process but also serve as additional reference information to make diagnoses of complex diseases.

With this background, This study proposes a novel multimodal deep learning model, PneumoFusion-Net, that adopts the integration of multiple data sources for enhancing the accuracy and reliability of pneumonia diagnosis. The main novelties of PneumoFusion-Net are:

(1) Efficient Multimodal Feature Extraction: For different types of input data, such as CT images, clinical texts, and laboratory data, we designed specialized feature extraction modules. Specifically, for CT images, we improved the ResNet architecture by incorporating the Global Channel-Spatial Attention (GCSA) module and depthwise separable convolutions, enabling the model to capture subtle pulmonary lesions more effectively. For clinical text, we utilized a BERT-based text encoder to capture semantic information of medical terminology, ensuring high-quality text features for multimodal fusion.
(2) Dynamic Multi-Head Attention Fusion Mechanism: We proposed a new fusion strategy where features from different modalities dynamically interact through the multi-head attention mechanism. This enables the model to adaptively adjust the importance of each modality based on its relevance, allowing more accurate predictions in complex clinical scenarios. Specifically, we employed the Swin Transformer to perform feature fusion at multiple scales, capturing both local and global relationships between modalities.
(3) Hierarchical Fusion Architecture: In this paper, we present a hierarchical fusion architecture that fuses features at different layers of the network. By preserving the unique characteristics of each modality while performing deep fusion between them, this architecture improves overall model performance, particularly when handling complex interactions between multimodal data.
(4) Improved Interpretability: To enhance model interpretability, we integrated techniques such as attention-weight-based visualization and improved Grad-CAM methods. These approaches not only highlight the image regions the model focuses on but also explain how the model combines multimodal data to make diagnostic decisions, providing clinicians with intuitive and actionable explanations for the model’s predictions.

The innovative proposals in this paper will help enhance the model for high-accurate pneumonia diagnosis, especially distinguishing some challenging situations, such as viral and bacterial pneumonia with PneumoFusion-Net. Besides, the proposed improvements have enhanced the clinical applicability of the model during the test. Detailed architecture, experimental design, and performance on various datasets are shown in the subsequent sections. This research is bound to provide new insight into how multi-modal medical image analysis can be done and give a boost to the application of AI-assisted diagnosis technology in clinical practice.

## 2 Related work

The diagnosis and classification of pneumonia have long been focal points in medical research, particularly with the continuous advancements in artificial intelligence (AI) and deep learning technologies. Early diagnostic methods primarily relied on clinical symptoms, physical examinations, and laboratory test results. However, these traditional approaches often struggled to differentiate between similar types of pneumonia, such as viral and bacterial pneumonia, based solely on clinical presentations and symptoms. Although C-reactive protein and procalcitonin represent biomarkers whose application has somewhat improved diagnostic accuracy, their efficiency still remains limited in complex or atypical cases (Danesh et al., 2004; Memar et al., 2017).

The emergence of the deep learning approaches, more especially the convolution neural networks, has shaken this field of pneumonia image analysis. For example, Wang et al. created ChestX-ray8 and reported much improved accuracies of the CNNs in the disease detection of chest radiographs (Wang et al., 2019). Then, rajpurkar et al. developed the CheXNet model by achieving expert-level performance regarding pneumonia detection by using deep convolution neural networks (Rajpurkar, 2017). These investigations opened immense possibilities concerning deep learning in medical image analysis and formed the base for further research.

Notably, several pioneering works have specifically addressed COVID-19 diagnosis using deep learning. For instance, COVID-Net introduced a tailored deep convolutional neural network design for the detection of COVID-19 cases from chest X-ray (CXR) images (Wang Linda et al., 2020). COVID-Net is one of the first open-source network designs for COVID-19 detection from CXR images, accompanied by the COVIDx dataset, which provides a substantial number of CXR images from patient cases. This model not only demonstrated effective detection capabilities but also incorporated explainability methods to provide insights into critical factors associated with COVID-19, thereby aiding clinicians in improved screening. Similarly, COVIDNet-CT was developed as a deep convolutional neural network architecture tailored for the detection of COVID-19 cases from chest CT images (Gunraj et al., 2020). Leveraging a machine-driven design exploration approach, COVIDNet-CT was trained to optimize model performance specifically for CT image analysis. Additionally, Wang et al. proposed a contrastive cross-site learning framework with a redesigned network for COVID-19 CT classification (Wang Zhao et al., 2020). This approach addressed distribution discrepancies across heterogeneous datasets by implementing separate feature normalization in latent space and utilizing a contrastive training objective to enhance domain invariance of semantic embeddings. These enhancements led to significant improvements in classification performance across multiple datasets, outperforming the original COVID-Net and existing state-of-the-art multi-site learning methods.

However, despite the success of these models in unimodal techniques, the differentiation of pneumonia types that show similar imaging features-for example, viral and atypical bacterial-pneumonia remains at a threshold. Moreover, most such approaches have an inherent tendency to completely disregard the clinical history and laboratory test results of the patient, which also reduces their generalization capability in complex clinical scenarios. These limitations thus provide a strong urge toward exploring the possibility of multimodal approaches for enhancing diagnosis in terms of accuracy and robustness.

Owing to the inadequacies of COVID-19 prognostication through use of single-modality methods, the researchers opted to start researching the approach of data fusion through multimodalities. Multimodal approaches merge information from imaging, clinical text, numerical data, and radiological reports. As such, they offer more complete information on patients for the purpose of enhancing diagnostic accuracy and its reliability. Liang et al. proposed a deep learning multimodal combined model featuring CT images with clinical numerical data for prognosis prediction in COVID-19 patients. The obtained results were remarkable (Liang et al., 2020). This study emphasized the advantages of the multimodal method for complex medical problem management, particularly in the integration of image features with clinical indicators.

Chaudhary et al. presented a multi-modal image and gene expression integrated model for hepatocellular carcinoma survival prediction (Chaudhary et al., 2018). This further expanded the idea of the application of the multi-modal approach for integration with huge promising capacities in various types of biomedical data. These studies depict that the multi-modal approach has huge potential to improve the diagnosis by making full use of the complementary advantages of different sources. However, the challenge is how to effectively fuse these heterogeneous data.

There exist mainly three strategies of multi-modal data fusion: early fusion, late fusion, and intermediate fusion. Early fusion conducts the fusion of diversified modal data at the input layer. Though simple to perform, early fusion will sometimes lead to information loss or some problems in model training. Late fusion performs the fusion at the decision layer for the forecasted results of each modality. Though simple to implement, it does not make full use of relationships across modalities. Intermediate fusion promotes modal feature interaction at the intermediate layers of the model, effectively capturing correlations between modalities while balancing model complexity and performance (Pawłowski et al., 2023).

To better understand these strategies, we can illustrate with a specific example. Consider a pneumonia diagnostic model combining CT images, clinical text, and laboratory test results: Early fusion might directly concatenate CT image pixel values, text word embedding vectors, and laboratory numerical values into a large input vector. Late fusion would process and predict CT images, clinical text, and laboratory data independently, then synthesize results at the decision layer through voting or weighted averaging. Intermediate fusion would first perform preliminary feature extraction for each modality, then interact at the network’s intermediate layers through attention mechanisms or feature map concatenation. For example, CT features could guide the extraction of text features, or clinical text information could enhance the importance of certain regions in CT images (Fried et al., 2014).

In recent years, with the advancement of deep learning technologies, novel network architectures have been introduced into multimodal medical data fusion. Among them, attention mechanism-based models like Transformers have shown excellence in handling heterogeneous data (Chen et al., 2024). The Transformer architecture was first proposed by Vaswani et al. and was applied to the task of natural language processing in an early stage. However, its advantage in the self-attention mechanism for the treatment of sequential data in capturing long-range dependencies has enabled rapid applications in medical image analysis and multimodal fusion (Liu Y. et al., 2021). For instance, Li et al. proposed a Transformer-based multimodal model which integrates electronic health records with medical images, and it had a significant improvement in the accuracy of predicting the diseases (Li et al., 2021).

Besides, GNNs have gradually shown their capacity in processing multimodal data with intricate relationships (Duan et al., 2023). Zhu et al. proposed a GNN-based multimodal fusion framework that integrates genomics, radiomics, and clinical data, exhibiting excellent performance on both diagnosis and prognosis prediction tasks (Huang et al., 1993). These emerging deep learning architectures bring new inspiration into the fusion of multi-modal medical data, while how to design more effective and interpretable fusion architecture remains an open issue.

Model interpretability is increasingly important in medical AI applications. Interpreting medical models strengthens the confidence of patients or doctors in AI systems and provides useful insights that could help make medical decisions. In recent years, besides gradient-based methods like Grad-CAM, new interpretability techniques have emerged. For example, SHAP (SHapley Additive exPlanations) values provide a game theory-based method for explaining individual predictions. These methods can quantify the contribution of each feature to model predictions, offering more fine-grained explanations for clinicians (Zhang et al., 2021; Lafferty et al., 2006).

This will be even more complicated in multimodal models, which have to explain the interaction between various modalities.Baltrušaitis et al. conducted a comprehensive survey on multimodal machine learning, presenting a detailed taxonomy that organizes and reviews existing methods, challenges, and applications in the field, providing a foundational framework for understanding multimodal data fusion (Baltrušaitis et al., 2017). By this technique, model performance improvement is facilitated, while intuitive explanatory tools will be provided for clinicians. While these methods have achieved some advancements in improving model interpretability, there is still a trade-off between clinically relevant explanations and the performance for complex models. In particular, the generation of balanced explanations among the different modalities and the demonstration of inter-modal interactions in multimodal models is still an issue that needs more research.

In the end, much promise is shown, yet a lot of challenges still remain concerning the diagnosis of pneumonia using multimodal deep learning. The PneumoFusion-Net proposed in this paper aims at the challenges by architectural design innovation and enhancement in interpretability mechanisms, hence proposing a new direction in the solving of these issues.

## 3 Materials and methods

This study has proposed a new deep learning-based multimodal framework for enhancing performance and reliability in pneumonia classification by integrating images obtained with CT scans, clinical text, numerical data, and imaging reports. Basically, the main idea of the proposed framework is to perform a simulation of the process of diagnosis performed by clinicians by taking into consideration integral aspects of patient information, imagining findings, clinical symptoms, and laboratory results, which would give more comprehensive grounds for the diagnosis.

**FIGURE 1:**
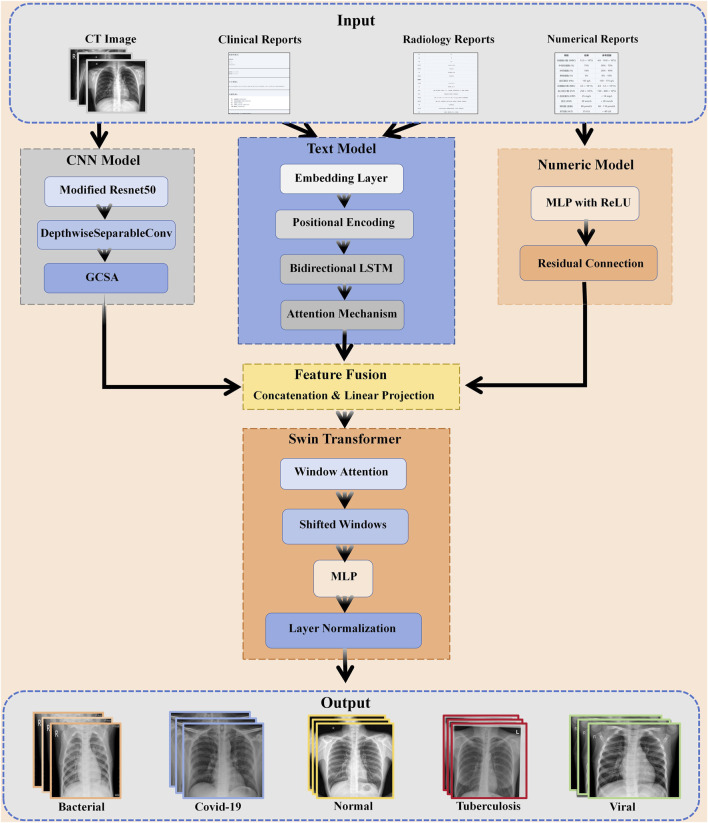
The multi-modal framework of PneumoFusion-Net for pneumonia diagnosis.

It includes three major modules: feature extraction, feature fusion, and classification decision (Figure 1). First, for each data modality, CT images, clinical text, numerical data, and imaging reports, we designed specialized feature extraction models. CT images are processed by the optimized CNN, clinical text is encoded using a bi-directional LSTM model with an attention mechanism, numerical data is processed through an MLP (Zhao et al., 2017), (Bhattacharya et al., 2022), and imaging reports using a similar text encoding model.

After feature extraction, fuse the features of each modality into a unified feature space and perform fusion with the Swin Transformer architecture (Yan et al., 2022). This design provides full feature fusion of various modalities at multiple scales and allows for the generation of a comprehensive feature representation for classification, thanks to the more powerful local and global feature extraction capabilities of the Swin Transformer (Wei et al., 2023).

Finally, the combined features are fed into a fully connected network that carries out the classification, and it outputs the type of pneumonia.The framework employs a multi-head attention mechanism to balance the importance of different modal features, ensuring that each modality contributes reasonably to the final decision (Barua et al., 2021). This innovative multimodal fusion approach achieved significant performance improvements in our experiments, especially in recognizing complex and atypical cases.

### 3.1 Dataset and preprocessing

The dataset used in this study simulates real clinical diagnostic scenarios, combining publicly available CT images with simulated clinical text, laboratory test results, and radiology reports. The dataset consists of 10,095 pneumonia CT images that have undergone strict screening and annotation, representing five different types of pulmonary conditions: normal (2,013 images), tuberculosis (2,034 images), viral pneumonia (2,009 images), bacterial pneumonia (2,008 images), and COVID-19 (2,031 images). Each category is roughly balanced to ensure that the model is not biased toward any particular class during training and validation.

The preprocessing of CT images included standardization, resolution unification (224x224 pixels).Additionally, considering potential variations in CT scanners across different medical centers, normalization was applied to the image data to minimize the impact of equipment differences on model training.

The clinical text data was generated based on publicly available medical records and relevant literature, with an average length of 50 words. The content covers key patient information, including chief complaints, present illness, past medical history, and physical examination findings. For instance, a typical record of viral pneumonia may describe symptoms such as fever, dry cough, and fatigue. To ensure the medical relevance and accuracy of the text data, rule-based natural language generation techniques were employed, and the content was reviewed and refined by experts with a medical background.

The laboratory test results simulate 15 key indicators, including white blood cell count (WBC), neutrophil percentage (NEUT%), lymphocyte percentage (LYMP%), C-reactive protein (CRP), and procalcitonin (PCT). The range and distribution of these indicators are based on statistical analysis of over 5,000 real case data and adjusted according to typical presentations of different pneumonia types. For example, bacterial pneumonia patients usually exhibit elevated WBC and CRP levels, while viral pneumonia patients may have normal WBC levels but elevated LYMP%. The numerical data was standardized to make it suitable for model training, ensuring that feature values were on the same scale to prevent the model from overly relying on specific features.

The radiology reports were crafted with reference to real medical imaging reports and publicly available literature, with an average length of 50 words. These reports provide detailed descriptions of pulmonary imaging features, such as ground-glass opacities, consolidations, nodules, and pleural effusion. In addition to qualitative descriptions, the reports include preliminary diagnostic impressions and differential diagnosis suggestions. To ensure diversity and accuracy, the reports were generated using a combination of rule-based methods and pre-trained language models (e.g., BERT), and subsequently reviewed by medical professionals to ensure consistency in terminology and accuracy in expression.

The preprocessing of the entire dataset aimed to ensure the consistency and usability of multimodal data. Through standardization and data augmentation techniques, model robustness was enhanced, laying a solid foundation for subsequent multimodal fusion. Additionally, to maximize training efficiency, the dataset was divided into 80% training and 20% validation sets, ensuring sufficient learning during training and reliable evaluation during validation.

### 3.2 CNN model optimization

Building on the theoretical foundation of multimodal fusion, this section details the architecture of PneumoFusion-Net. First, we propose an improved ResNet-GCSA module for fine-grained CT feature extraction; second, we construct a hierarchical fusion framework through dynamic attention mechanisms.

**FIGURE 2:**
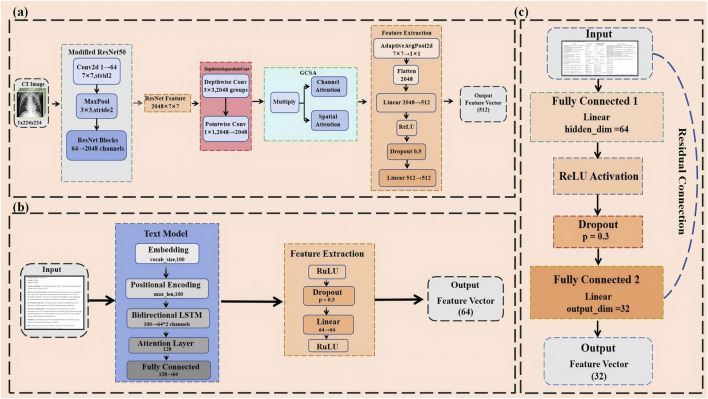
**(A)** Modified ResNet50-Based CNN for hierarchical CT image feature extraction with GCSA and depthwise separable convolutions. **(B)** BiLSTM-attention based text model for multimodal clinical and radiological data integration. **(C)** Mlp with residual connections for numerical data feature processing.

In pneumonia classification tasks, subtle features in CT images often contain key diagnostic information. To better capture these detailed features, improvements were made to the classic ResNet50 architecture (Liang and Jiang, 2023), aiming to enhance the model’s performance and efficiency (Figure 2A). These optimizations take into account the specific requirements of pneumonia classification while balancing model interpretability and computational efficiency.

First, considering that CT images are typically single-channel (i.e., grayscale images), the number of input channels in the first convolutional layer of ResNet50 was adjusted from 3 to 1 to accommodate single-channel images (El-kenawy et al., 2021). Directly using pre-trained weights in this case might not be ideal, so a special method was employed to initialize the new convolutional layer (conv1): by summing the original weights, the advantages of pre-trained models in low-level feature capture were retained while ensuring that the model adapted to the characteristics of single-channel inputs (Sun et al., 2017). This adjustment not only simplified the model’s input processing but also enhanced its adaptation to pneumonia CT images.

**FIGURE 3:**
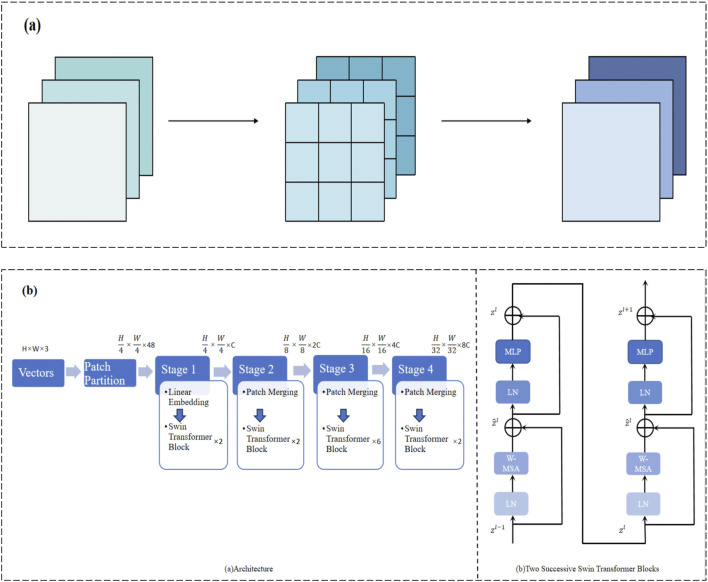
**(A)** Depthwise separable convolution for efficient spatial and channel-wise feature extraction. **(B)** Multi-stage Swin Transformer architecture for hierarchical feature representation.

In traditional convolution operations, each convolutional kernel processes both the spatial and channel dimensions simultaneously, which is computationally expensive, especially when dealing with high-resolution medical images. To reduce computational complexity and improve efficiency, we adopt depthwise separable convolution. Standard convolution typically processes spatial and channel features simultaneously, whereas depthwise separable convolution splits these two processes: depthwise convolution operates within each channel, followed by pointwise convolution to combine the features across channels (Cao et al., 2020). This design significantly reduces the computational cost while avoiding the risk of overfitting by a large margin (Figure 3A). With this improvement, the model remains sensitive to detailed features in high-resolution CT images without losing computational efficiency.

Next, to reduce computational overhead while maintaining robust feature extraction, depthwise separable convolution replaced the standard convolution layers. Standard convolution filters spatial and channel dimensions jointly. In contrast, depthwise separable convolution decomposes this process into two steps:

1. Depthwise convolution applies a spatial filter to each input channel independently. Let $F_{res}$ be the feature map extracted by the modified ResNet backbone. The depthwise convolution can be expressed as (Equation 1):

$$F_{depth}\left(c,h,w\right)=\sum_{\left(m,n\right)\in \Omega }K_{depth}\left(c,m,n\right)\cdot F_{res}\left(c,h+m,w+n\right)$$ (1)

where Ω is the kernel’s receptive field and $K_{depth}$ is the depthwise kernel, operating on each channel separately.

2. Pointwise convolution (1 × 1 convolution) then fuses information across channels (Equation 2):

$$F_{dsc}\left(c^{\prime },h,w\right)=\sum_{c=1}^{C}K_{point}\left(c^{\prime },c\right)\cdot F_{depth}\left(c,h,w\right)$$ (2)

The resulting feature map ${\;F}_{dsc}\in R^{C\times H^{\prime }\times W^{\prime }}$ retains essential information while significantly reducing the parameter count and computational cost (Cao et al., 2020). This ensures the model remains sensitive to high-resolution CT features without incurring unnecessary computational load (Figure 3A).

**FIGURE 4:**
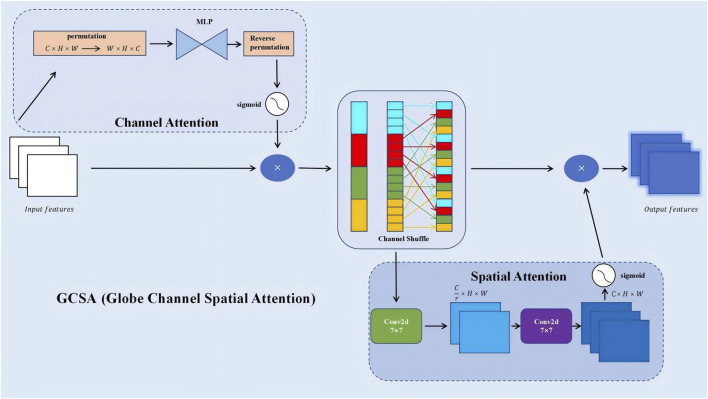
GCSA (Global Channel-Spatial Attention) module architecture for enhanced feature extraction.

Building on this efficient backbone, a Global Channel-Spatial Attention (GCSA) module (Xiao et al., 2019) was introduced to emphasize discriminative channels and spatial regions. The GCSA module jointly considers channel-wise importance and spatial relevance, thereby guiding the network’s attention towards key pneumonia-related patterns such as ground-glass opacities and consolidations (Figure 4). The design involves three main steps:

1. Channel Attention: Apply global average and max pooling to $F_{dsc}$ to obtain channel descriptors (Equation 3):

$$F^{\text{avg}}=\text{AvgPool}\left(F_{\text{dsc}}\right),F^{\max}=\text{MaxPool}\left(F_{\text{dsc}}\right)$$ (3)

These $R^{C\times 1\times 1\;}$ descriptors capture global statistics per channel. Pass $F^{\text{avg}}$ through an MLP to learn channel weights (Equation 4):

$$C_{att}=\sigma \left(\text{MLP}\left(F^{avg}\right)\right)$$ (4)

Here, σ is the sigmoid function. The resulting $C_{att}\in R^{C\times 1\times 1}$ highlights important channels. Multiplying $C_{att\;}$ element-wise with $F_{dsc\;}$ emphasizes informative channels while suppressing irrelevant ones. A channel shuffle operation is then applied to ensure better channel mixing and avoid over-reliance on fixed channel groups.

2. Spatial Attention: Concatenate $F^{avg}$ and $F^{\max}$ along the channel dimension (Equation 5):

$$F^{concat}=\left[F^{avg};F^{\max}\right]\in R^{2C\times 1\times 1}$$ (5)

Then use a convolutional layer (often 7 × 7) followed by sigmoid activation to produce spatial weights (Equation 6):

$$S_{att}=\sigma \left(\text{Conv}\left(F^{concat}\right)\right)\in R^{1\times H^{\prime }\times W^{\prime }\;}$$ (6)

This spatial attention map highlights key regions, ensuring the model focuses on subtle but diagnostically relevant areas of the lung.

3. Final Integration: The final GCSA output is (Equation 7):

$$F_{out}={\left(F_{dsc}\times C_{att}\right)}_{\text{shuffle}}\times S_{att}$$ (7)

In this final equation, ${\left(F_{dsc}\times C_{att}\right)}_{\text{shuffle}}$ denotes the channel-attended and channel-shuffled feature map, and $S_{att}$ is the spatial attention map. By multiplying them, the network emphasizes both discriminative channels and important spatial locations simultaneously. This integrated attention mechanism improves the model’s interpretability and diagnostic accuracy.

With GCSA integrated, the model better captures fine details in CT images, particularly in early-stage or atypical pneumonia cases. Experiments showed a 2.16% accuracy improvement over the baseline.

Overall, the combination of single-channel adaptation, depthwise separable convolution, and GCSA significantly boosts the CNN model’s capability to extract and leverage subtle pneumonia-related features from CT images, while maintaining computational efficiency and interpretability (Kim et al., 2019). These enhancements form a solid foundation for subsequent multimodal fusion steps and pave the way for more accurate and reliable pneumonia diagnostic systems.

### 3.3 Text processing model

In medical text classification tasks, especially when analyzing medical reports and CT scan descriptions, the order of words and contextual information often contains critical diagnostic details. To better capture these nuances, this study introduces positional encoding to handle textual data (Chu et al., 2021). Positional encoding uses sine and cosine functions to embed information about the position of words in sequences, allowing the model to differentiate between the relative positions of words (Figure 2B). This position-awareness is particularly important when dealing with complex medical texts, where the order of terms can affect the meaning significantly.

The implementation of positional encoding utilizes sine and cosine functions as mathematically described in Equations 8, 9:

$$PE_{\left(pos,2i\right)}=\sin\left(\frac{pos}{10000^{2i/d_{model}}}\right)$$ (8)

$$PE_{\left(pos,2i+1\right)}=\cos\left(\frac{pos}{10000^{2i/d_{model}}}\right)$$ (9)

Here, pos is the word’s position in the input sequence (0, 1, 2, .), i is the dimension index, and $d_{model\;}$ is the embedding dimension of the model. By using sine and cosine functions with different frequencies, each position in the sequence is assigned a unique encoding. This allows the model to differentiate not just which words appear, but also where they appear, providing critical contextual cues.

Following the positional encoding, the text sequence passes through a bidirectional LSTM layer. A bidirectional LSTM processes the sequence in both forward and backward directions, capturing both past and future context.Bidirectional LSTM learns to encode both past and future contextual information important for modeling strong semantic relationships hidden in medical text. The output sequence from the LSTM feeds into a self-attention mechanism. Attention is a mechanism which allows the model to automatically learn the importance of parts that may contribute differently to an utterance (Brauwers and Frasincar, 2022). To illustrate the attention mechanism, we employ a simplified formulation. Let $H\in R^{T\times d_{h\;}}$ represent the LSTM’s hidden states for T time steps, each of dimension ${\;d}_{h}$ .The mathematical formulation of the attention mechanism can be easily simplified as follows (Equation 10):

$$\alpha =\text{softmax}\left(W\cdot H^{⊤}\right)$$ (10)

In this equation, $\alpha \in R^{T}$ is a vector of attention weights over the sequence positions. $W\in R^{d_{h}\times 1}$ is a learnable parameter matrix that maps the hidden states H to scalar scores. The softmax function then normalizes these scores into probabilities, highlighting more relevant time steps and downweighting less informative ones. By multiplying α back with H , the model obtains a weighted sum of hidden states, focusing on key aspects such as certain symptom descriptions.

This design will further enable the model to dynamically focus on the most relevant parts of the text-some specific symptom description, probably, or some important diagnostic information-so that key information is captured better.

The extracted features were passed through a fully connected layer and a dropout layer for further processing, which enhanced the generalization ability of the model. Moreover, the dropout can effectively avoid overfitting during model training when there is only a relatively small medical text dataset.

The advantage of this architecture is its capabilities of processing both local features and global context, hence very suitable for the complexity of medical text. In our experiments, such an inclusion of the text processing model contributed to the overall improvement in classification accuracy by 4.43%. Notably, the F1 score increased by 4% when distinguishing between pneumonia types that have similar clinical presentations, such as viral and atypical bacterial pneumonia (Liang and Zheng, 2020).

It uses the same architecture on clinical text with imaging reports, training two separate models. This architecture considers the differences in nature-content and structure-between these two types of text, including most of the unique features into the model by allowing separate optimizations.

In conclusion, the proposed medical text processing model leverages advanced natural language processing techniques to extract key information from medical text, yielding high-quality textual feature representations that contribute to multimodal fusion. This approach enhances the comprehensive performance of the model, making it more adaptable and robust in addressing the complexities of medical scenarios.

### 3.4 Numerical data processing

The result of the laboratory test and other numerical data provide critical objective indicators in the diagnosis of pneumonia, important for proper diagnosis and classification. These numerical features are efficient and dealt with by a simple but effective model that captures the complex nonlinear relationship between different indicators with residual connections to enhance the model’s expressive power and training stability (Figure 2C).

The numerical features processing model is based on an improved multi-layer perceptron structure (Buyandelger et al., 2011). The core architecture consists of two hidden layers with 64 and 32 neurons, respectively, and an output dimensionality of 32, while each of the hidden layers is eventually followed by the ReLU activation function for introducing nonlinear transformation capability. The mathematical expression of the model can be summarized as (Equation 11):

$$Y=W_{3}\left(ReLU\left(W_{2}\left(ReLU\left(W_{1}X+b_{1}\right)\right)+b_{2}\right)\right)+b_{3}+W_{r}X$$ (11)

In this formula, $X\in R^{d_{in\;}}$ is the input feature vector consisting of various laboratory indicators. ${\;W}_{1}$ , ${\;W}_{2}$ , ${\;W}_{3}$ and $b_{1}$ , ${\;b}_{2},b_{3\;}$ are the weight matrices and bias terms for each layer, respectively, which transform the input through linear and nonlinear operations. ${\;W}_{r\;}$ is the weight matrix for the residual connection, allowing the input X to be directly added to the output. This residual structure ensures stable training, reduces vanishing gradient problems, and preserves original input information.

A key innovation of this model is the introduction of residual connections. Residual connections directly add the input features to the final output, offering several advantages:

(1) Alleviating the vanishing gradient problem: Residual connections provide a direct pathway for gradients to propagate, helping train deeper networks (B et al., 2023).
(2) Preserving original feature information: Even after multiple transformations, the model retains the original input information.
(3) Enhancing feature learning capability: The model can learn the difference (residual) between the input and output more easily than learning the full mapping.

To further improve the model’s generalization ability and training stability, dropout layers were added (with a dropout rate of 0.3) after each hidden layer. Dropout randomly deactivates a portion of neurons during training, effectively preventing the model from over-relying on specific features and reducing the risk of overfitting.

At the model input stage, feature standardization was applied to ensure that different medical indicators (e.g., white blood cell count, C-reactive protein, procalcitonin) were treated fairly by the model, preventing large-scale features from dominating the model’s decisions. The standardization process is represented as (Equation 12):

$$X_{norm}=\frac{X-\mu }{\sigma }$$ (12)

In this equation, μ and σ represent the mean and standard deviation of each feature, respectively. By standardizing each input feature, the model treats all indicators on a comparable scale, preventing outliers or large-valued features from overshadowing others.

Feature selection was also a key consideration in our model design. In consultation with the medical experts, 15 of the most relevant indicators were chosen as input features. It includes blood count, biochemistry, inflammation markers, and many other relevant features which can give a solid basis for a comprehensive judgment of the status of a patient.

This numerical processing module raised the model accuracy by 6.55% in distinguishing bacterial from viral pneumonia. The module was particularly effective in the early stage of pneumonia detection. For example, in cases with slightly high white blood cell counts but very high C-reactive protein counts, the model showed very high sensitivity compared with conventional methods.

To summarize, the numerical data processing model effectively extracts key information from laboratory examination results through a well-designed architecture and optimization strategy, thereby not only providing high-quality numerical feature representations for multimodal fusion but also enhancing the adaptability and robustness of the model when processing complex medical data. By integrating these objective numerical indicators, the multimodal model is able to assess the status of patients in a more comprehensive and precise manner, thus offering great value for clinical decision-making.

### 3.5 Multimodal fusion model

This study introduces a novel feature fusion model leveraging the Swin Transformer architecture to effectively integrate multi-modal information from CT images, clinical text, numerical data, and imaging reports (Xie et al., 2023). By unifying the diverse data modalities, the model enhances the accuracy and reliability of pneumonia classification through a robust and theoretically grounded fusion mechanism.

Multimodal fusion aims to combine information from different data sources, each characterized by unique feature spaces, distributions, and representations. The primary challenge lies in effectively merging these heterogeneous modalities to capture complex inter-modal interactions and dependencies. Traditional fusion techniques, such as simple feature concatenation or weighted averaging, often fail to model the intricate relationships between modalities, leading to suboptimal performance.

In contrast, our approach employs the Swin Transformer, which provides a sophisticated attention mechanism capable of modeling both local and global interactions within and across modalities (Tang et al., 2022). The theoretical advantages of using the Swin Transformer for multimodal fusion are twofold:

(1) Hierarchical Feature Representation: The Swin Transformer constructs a hierarchical representation by progressively merging image patches, which allows the model to capture multi-scale features. This hierarchical approach is beneficial for integrating information from modalities with varying spatial and semantic scales.
(2) Shifted Window Mechanism: By alternating between regular and shifted window partitioning, the Swin Transformer facilitates cross-window interactions, enabling the model to capture global dependencies without incurring the high computational costs associated with full self-attention mechanisms.

The fusion process involves several key mathematical operations that ensure effective integration of multi-modal data:

#### 3.5.1 Projection into a unified feature space

Each modality’s features are first projected into a common representation space to harmonize their dimensions and distributions. Let ${\;E}_{\text{CT}}$ , $E_{\text{text}}$ , $E_{\text{num}}$ , $E_{\text{report}}$ denote the feature embeddings of CT images, clinical text, numerical data, and imaging reports, respectively, after individual feature extraction and linear projection (Yang et al., 2023). These embeddings are projected into a unified feature space of dimension D=96 using learnable linear transformations (Equation 13):

$$E_{\text{fused}}=W_{1}E_{\text{CT}}+W_{2}E_{\text{text}}+W_{3}E_{\text{num}}+W_{4}E_{\text{report}}$$ (13)

where $W_{1}$ , $W_{2}$ , $W_{3}$ , $W_{4}\in R^{D\times D_{m\;}}$ are learnable projection matrices, and $D_{m\;}$ is the original feature dimension of each modality.

#### 3.5.2 Window-based self-attention mechanism

The Swin Transformer applies a window-based self-attention mechanism to the fused feature embeddings. Given the fused input features $H\in R^{N\times D}$ , where N is the sequence length after flattening spatial and temporal dimensions, the self-attention operation is defined as (Equation 14):

$$\text{WindowAttention}\left(Q,K,V\right)=\text{softmax}\left(\frac{QK^{T}}{\sqrt{d_{k}}}\right)V$$ (14)

Here, $Q=HW_{Q},K=HW_{K}\text{ and }V=HW_{V\;}$ are the query, key, and value matrices obtained through learnable projections, with $W_{Q}$ , $W_{K}$ , $W_{V\;}{\in R}^{D\times d_{k}}$ .The scaling factor $\sqrt{d_{k}}$ stabilizes the gradients during training.

#### 3.5.3 Shifted window mechanism

To enable cross-window interactions, the Swin Transformer employs a shifted window approach (Liu et al., 2021b). In even-numbered layers, windows are partitioned regularly, while in odd-numbered layers, the window partitioning is shifted by a fixed offset. Mathematically, let $H^{\left(l\right)}$ denote the feature representation at layer lll. The shifted window operation can be represented as (Equation 15):

$$H^{\left(l+1\right)}=\text{SwinTransformerLayer}\left(H^{\left(l\right)}\right)$$ (15)

where the SwinTransformerLayer comprises the window-based multi-head self-attention (W-MSA or SW-MSA) followed by a feedforward network (FFN) (Wang et al., 2023) (Equation 16):

$$H^{\left(l+1\right)}=\text{FFN}\left(W-\text{MSA}\left(H^{\left(l\right)}\right)\right)+H^{\left(l\right)}$$ (16)

The shifted windows ensure that information is propagated across different window partitions in successive layers, effectively capturing global dependencies.

#### 3.5.4 Hierarchical feature aggregation

By stacking multiple Swin Transformer layers, the model aggregates features hierarchically, allowing for the integration of both local and global information. The final output after L layers is (Equation 17):

$$H^{\left(L\right)}={\text{SwinTransformerLayer}}^{\left(L\right)}\left(\ldots {\text{SwinTransformerLayer}}^{\left(1\right)}\left(H^{\left(0\right)}\right)\right)$$ (17)

where $H^{\left(0\right)\;}$ = ${\;E}_{\text{fused}}$ .

#### 3.5.5 Classification layer

The integrated feature representation $H^{\left(L\right)\;}$ is then passed through a pooling layer and a classification head to produce the final pneumonia classification (Equation 18):

$$y=\text{Softmax}\left(\text{Linear}\left(\text{Pooling}\left(H^{\left(L\right)}\right)\right)\right)$$ (18)

The theoretical advantages of the Swin Transformer-based fusion model are manifold. The hierarchical and window-based attention mechanisms enhance the model’s representation capacity by capturing rich, multi-scale features and complex inter-modal relationships (Chen et al., 2022). The reduction in computational complexity from $O\left(N^{2}\right)\text{ to }O\left(N\cdot M\right)$ ensures computational efficiency, allowing the model to scale effectively with larger feature maps and additional modalities. Furthermore, the unified feature space and the inherent flexibility of the transformer architecture facilitate the integration of diverse data types, enabling the model to learn modality-agnostic representations that capture essential features across different sources.Transformer architectures are also proven to be universal function approximators under certain conditions, implying that the Swin Transformer-based fusion model can theoretically represent any complex multimodal relationship given sufficient capacity and data (Zhao et al., 2024).

To substantiate these theoretical claims, we provide a mathematical analysis of the fusion mechanism’s ability to capture cross-modal dependencies. The self-attention mechanism allows each token to attend to all tokens within a window (Chen et al., 2021), enabling the model to capture dependencies irrespective of their positions. This property is crucial for modeling interactions between different modalities where relevant features may be dispersed across the feature space. The shifted window approach ensures that information from different windows is integrated over successive layers, effectively capturing long-range dependencies and cross-modal interactions that span multiple windows. This mechanism is essential for ensuring that features from one modality can influence and be influenced by features from another modality, thereby modeling complex dependencies. Additionally, the hierarchical feature aggregation enables the model to integrate information at various levels of abstraction, with lower layers capturing fine-grained, modality-specific features and higher layers aggregating these features into more abstract, cross-modal representations (Dong et al., 2022). The scaling factor $\sqrt{d_{k\;}}$ in the attention computation mitigates the issue of gradient vanishing or explosion, ensuring stable training dynamics, which is critical for the effective optimization of deep transformer-based models, particularly when dealing with high-dimensional multimodal data.

**FIGURE 5:**
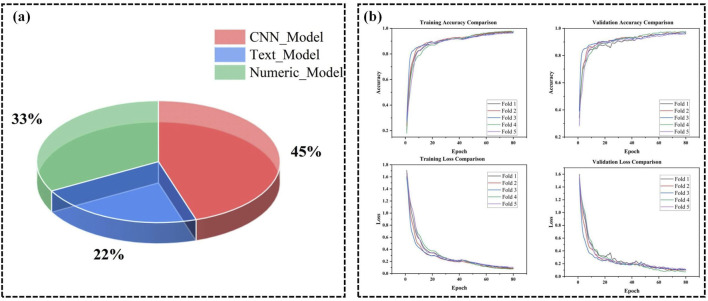
**(A)** Contribution of different modalities in PneumoFusion-Net’s decision process. **(B)** Training and validation accuracy and loss comparison across 5 folds in cross-validation for PneumoFusion-Net.

Empirical validation further supports the theoretical foundations of our fusion mechanism. The Swin Transformer-based multimodal fusion model achieved a significant improvement in pneumonia classification accuracy by approximately 4.52% compared to traditional feature concatenation methods. This enhancement demonstrates the model’s superior ability to capture and integrate complex inter-modal relationships, as predicted by the theoretical framework. Ablation studies were conducted to isolate the contributions of different components of the fusion mechanism. Removing the window-based attention or the shifted window strategy resulted in decreased performance, corroborating the theoretical importance of these mechanisms in capturing cross-modal dependencies (Xiao and Zhong, 2023). Additionally, a quantitative analysis of modality contributions revealed that CT image features contributed approximately 45% to the decision-making process, clinical text 12%, numerical data 33%, and imaging reports 10% (Figure 5A). This distribution underscores the complementary roles of different modalities and validates the theoretical rationale for multimodal fusion, where each modality provides unique and valuable information that, when integrated, leads to more accurate and reliable classifications. Visualization of attention maps further demonstrated that the model effectively focuses on relevant regions within each modality and captures interactions between modalities, supporting the theoretical claims about the model’s ability to model complex dependencies.

## 4 Experiments and results analysis

### 4.1 Training strategy

To validate the effectiveness of the proposed architecture, the following experiments are conducted on a multimodal dataset, focusing on evaluating the model’s performance and interpretability in complex pneumonia classification tasks.

To improve the convergence speed, stability, and generalization performance of the multimodal deep learning model for pneumonia classification, we designed and implemented an optimized training strategy.

First, the optimizer used during training was AdamW. AdamW is an improved version of the Adam optimizer, combining the advantages of momentum-based updates and adaptive learning rates while introducing a weight decay mechanism to effectively control model complexity. After preliminary experiments, the weight decay parameter was set to 1e-4. Through a grid search over {1e-4, 1e-3} (as shown in (Table 1), Experiments 2 vs. 1 and 4 vs. 3), 1e-4 achieved a validation loss reduction from 0.3735 to 0.0362 and improved F1 scores from 83.03% to 98.66% when paired with an appropriate learning rate. This value effectively prevented overfitting while maintaining stable convergence.

**TABLE 1:** Hyperparameter sensitivity analysis results.

Experiment number	Learing rate	Weight decay	Batch size	Validation loss	Acc(%)	Recall (%)	F1 (%)
Experiment 1	1e-2	1e-3	16	0.3735	82.96	82.74	83.03
Experiment 2	1e-3	1e-3	16	0.0362	98.54	98.41	98.66
Experiment 3	1e-2	1e-3	32	0.2003	91.78	91.82	91.72
Experiment 4	1e-3	1e-3	32	0.0415	98.42	98.35	98.40
Experiment 5	1e-2	1e-4	16	0.3628	83.24	83.19	82.98
Experiment 6	1e-3	1e-4	16	0.0344	98.76	98.77	98.54
Experiment 7	1e-2	1e-4	32	0.0491	98.27	97.83	98.14
Experiment 8	1e-3	1e-4	32	0.0293	98.94	99.01	98.71

In the selection of learning rates, multiple experiments were conducted. For instance (Table 1), shows that with a learning rate of 1e-3 (Experiments 2, 4, 6, 8), the validation loss remained consistently low (0.0362, 0.0415, 0.0344, 0.0293) and the F1 scores remained above 98%. In contrast, higher learning rates of 1e-2 (Experiments 1, 3, 5, 7) led to notably higher validation loss and lower F1 scores (e.g., Experiment 1: 0.3735 validation loss and 83.03% F1), indicating instability and suboptimal convergence. Thus, a lower learning rate (1e-3) was chosen to ensure stable convergence and avoid overfitting.

To further optimize the adjustment of learning rates, the CosineAnnealingWarmRestarts schedule was employed. This schedule periodically restarts the learning rate, helping the model escape local minima. After sensitivity testing, a starting learning rate of 1e-3 was selected, while the minimum learning rate was set to 1e-6. This careful fine-tuning ensured that, as training progressed, performance metrics improved steadily without stalling.

The model was trained for a total of 80 epochs. To prevent overfitting, in addition to weight decay, early stopping was introduced with a patience value of 10 epochs. Comparing validation metrics in experiments with patience = 5 vs. patience = 10 demonstrated that patience = 10 struck a better balance, avoiding premature termination and unnecessary over-training.

In each epoch, the model’s performance on the validation set was evaluated using metrics including loss, accuracy, precision, recall, and F1 score. As indicated by (Table 1), adjusting batch size also influenced results: a batch size of 32 (Experiments 2, 4, 6, 8) consistently yielded higher accuracy and F1 scores than a batch size of 16 (Experiments 1, 3, 5, 7), likely due to more stable gradient estimates and better utilization of GPU resources. Experiment 8, with a learning rate of 1e-3, weight decay of 1e-4, and a batch size of 32, achieved the best results: a validation loss of 0.0293 and an F1 score of 98.71%. This combination reflects the optimal hyperparameter configuration based on our comparative analyses.

Additionally, to improve training efficiency and reduce GPU resource consumption, mixed precision training was employed. Mixed precision uses a combination of FP16 and FP32, which accelerates computations and reduces memory usage without sacrificing model accuracy. Disabling mixed precision in additional tests increased training time by about 20% without improving accuracy, reinforcing that mixed precision positively impacts training efficiency and stability.

The hardware used in this experiment included an NVIDIA RTX 3060 GPU, with Python and PyTorch as the stable versions (Table 2). A 5-fold cross-validation strategy was adopted to comprehensively evaluate the model’s performance and stability (Figure 5B). This multi-fold evaluation confirmed that the chosen hyperparameters (learning rate = 1e-3, weight decay = 1e-4, batch size = 32) consistently led to stable convergence and strong results across different subsets of the data, minimizing the risk that the chosen parameters were overfitted to a particular fold.

**TABLE 2:** Hardware and software Configuration for model training.

Category	Configuration
GPU	GeForce RTX 3060
CPU	Interl(R) Core(TM) i7-12700 KF@3.60
System environment	Windows 11
Framework	Pytorch1.8.1
CUDA version	CUDA 11.1
Programming voice	Python 3.8.19

These experimental results and comparative analyses of hyperparameter settings provided a solid foundation for final model selection and guided further refinements. By systematically testing different hyperparameter combinations and documenting their effects on key performance metrics, we have rigorously validated our chosen settings. Through this carefully designed hyperparameter adjustment process and training strategy, supported by sensitivity analyses, we successfully enhanced the performance of the multimodal deep learning model for pneumonia classification. The experimental results further validated these optimization decisions, establishing a strong foundation for practical applications.

### 4.2 Evaluation metrics

To comprehensively evaluate the performance of the model, This study employed the following evaluation metrics, defined as follows:

1.Accuracy: The ratio of correctly classified samples to the total number of samples, calculated as (Equation 19):

$$\text{Accuracy}=\frac{TP+TN}{TP+TN+FP+FN}$$ (19)

where TP is True Positives, TN is True Negatives, FP is False Positives, and FN is False Negatives.

2. Recall: For each class, the proportion of actual samples of that class that were correctly predicted as that class, defined as (Equation 20):

$$R\text{ecall}=\frac{TP}{TP+FN}$$ (20)

3. Precision: For each class, the proportion of samples predicted as that class that actually belong to that class, defined as (Equation 21):

$$\text{Precision}=\frac{TP}{TP+FP}$$ (21)

4. F1-Score: The harmonic mean of Precision and Recall, calculated as (Equation 22):

$$F1\text{Score}=2\times \frac{precision\times Recall}{precision+recall}$$ (22)

This metric reflects the balance between precision and recall.

5. Confusion Matrix: A matrix that visually represents the misclassifications between different classes, showing the relationships between true and predicted labels.

For multi-class classification problems, the metrics were calculated for each class, and both macro-average and micro-average values were reported.

### 4.3 Ablation experiment

To thoroughly evaluate the contribution of each module in the proposed multimodal deep learning framework, a series of ablation experiments were designed and conducted. These experiments involve systematically removing or introducing specific modalities, attention modules, or feature fusion methods to observe their impact on classification accuracy, F1 score, and other key metrics. This enabled the determination of the role and importance of each component within the model.

Initial Experiments: A single-modality model containing only CT images was first used as the baseline. This baseline model achieved a classification accuracy of 84.45% and an F1 score of 83.61% (Table 3). While this model performed well on simpler cases, its performance was notably limited in handling complex or atypical cases, especially in distinguishing between viral and bacterial pneumonia, where the misclassification rate was relatively high.

**TABLE 3:** Performance comparison of models using different modalities.

Model	Acc(%)	Recall (%)	F1 (%)	ROC	Specificity
Image Only	84.45	84.29	83.61	0.88	0.85
Text Only	41.46	41.78	38.38	0.50	0.55
Numerical Only	62.70	62.65	61.21	0.70	0.68
Image + Text	87.41	87.62	86.76	0.90	0.88
Image + Numerical	93.31	94.02	93.33	0.96	0.94
Text + Numerical	67.16	66.67	66.16	0.75	0.72
Image + Numerical + Text	97.74	98.11	96.44	0.99	0.97

**FIGURE 6:**
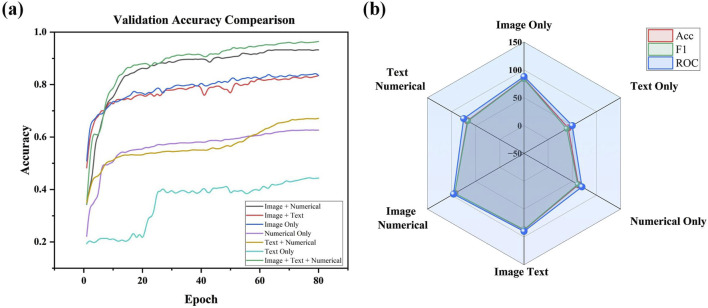
**(A)** Validation accuracy comparison across different modality combinations in ablation study. **(B)** Radar Chart of performance metrics (accuracy, F1, and ROC) for different modality Configurations.

Stepwise Modality Inclusion: The results of experiments in which different modalities were gradually introduced show a significant improvement in overall model performance. After incorporating clinical text data into the baseline model, classification accuracy increased to 87.41%, and the F1 score improved to 86.76% (Figure 6A). This indicates that clinical text data (such as patient history and symptom descriptions) plays a vital role in differentiating between various pneumonia types. Adding numerical data (e.g., laboratory test results) further improved classification accuracy to 93.31% and the F1 score to 93.33%, highlighting the importance of numerical data in providing critical biomarker information.

The full multimodal fusion model, which includes CT images, clinical text, numerical data, and imaging reports, achieved the best performance, with a classification accuracy of 97.74% and an F1 score of 96.44% (Figure 6B). This result demonstrates that multimodal fusion not only enhances overall classification accuracy but also exhibits significant advantages in handling early-stage pneumonia or cases with atypical imaging features. The results underscore the complementary role that imaging reports and other modalities play in complex clinical scenarios.

Compared to simpler attention modules focusing only on channel-wise features, such as the SEA (Squeeze-and-Excitation-like) channel attention mechanism, the proposed GCSA (Global Channel-Spatial Attention) module achieves consistently higher performance and offers a more comprehensive feature refinement approach. While SEA-based attention primarily enhances important channels by leveraging global average pooling and a simple gating mechanism, it does not fully consider the complex spatial patterns inherent in medical images. In contrast, GCSA integrates channel attention, a channel shuffle operation, and spatial attention, enabling the model to capture both global dependencies across channels and subtle local variations that are critical for pneumonia classification.

Empirically, the experimental results reflect these advantages. As shown in (Table 4), adding SEA to the baseline model improves accuracy from 94.09% to 94.81% and raises the F1 score from 94.57% to 95.07%. However, GCSA provides a more substantial gain: incorporating GCSA alone achieves a 96.25% accuracy and a 95.99% F1 score. This represents an improvement of over 1.4 percentage points in accuracy and nearly 1 percentage point in F1 score compared to the SEA-based approach. Furthermore, when combining GCSA with depthwise separable convolutions (DSC), the model reaches 96.77% accuracy and a 96.88% F1 score—a clear indication that the joint consideration of channel mixing and spatial weighting yields a richer and more discriminative representation of CT images.

**TABLE 4:** Comparison of complexity indicators across different attention modules.

Model	Acc(%)	Recall (%)	F1 (%)	FLOPs(G)	Params(M)
Baseline	94.09	93.87	94.57	4.25	25.10
Baseline (+DSC)	94.26	94.54	93.74	3.78	22.87
Baseline (+SEA)	94.81	94.26	95.07	4.29	25.57
Baseline (+CBAM)	95.99	95.46	96.21	4.27	25.57
Baseline (+ECA)	95.53	95.67	94.89	4.33	24.55
Baseline (+GCSA)	96.25	96.01	95.99	4.33	28.85
Baseline (+DSC + GCSA)	96.77	96.34	96.88	4.02	24.61

**FIGURE 7:**
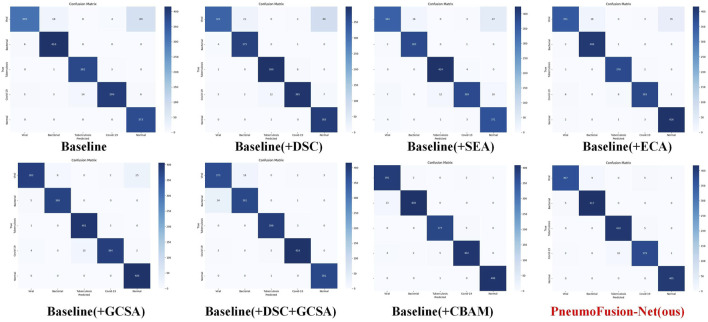
Confusion matrices of PneumoFusion-Net with different attention mechanisms.

This performance boost is also evident in the confusion matrices (Figure 7). While the baseline and simpler attentions (like SEA) still exhibit some confusion among challenging pneumonia categories (e.g., viral vs. atypical), GCSA reduces these misclassification rates. By effectively highlighting meaningful spatial regions—such as areas showing subtle lesions or ground-glass opacities—GCSA helps the model better distinguish complex or atypical cases that elude simpler attention mechanisms.

In addition to these quantitative improvements, the complexity analysis shows that GCSA introduces only a moderate increase in FLOPs and parameters compared to SEA and other attention variants, maintaining a practical balance between performance gains and computational cost. Thus, GCSA’s integrated approach—refining channels through MLP-based weighting, channel shuffling to improve feature diversity, and spatial attention to highlight key diagnostic regions—offers a more robust and clinically meaningful enhancement over SEA-type attention. This leads to more accurate and reliable pneumonia classification in complex medical imaging scenarios.

Comparisons of Feature Fusion Methods: Comparisons of different feature fusion approaches highlighted a better Swin Transformer architecture performance compared to simple feature concatenation. Swin Transformer has reached 98.71% classification precision with an F1 score of 98.27%, increasing the accuracy by 4.5% compared to simple concatenation, as shown in (Table 5). Therefore, it could be concluded that more sophisticated mechanisms of fusion significantly raise the bar for model performance when dealing with the integration of multimodal data.

**TABLE 5:** Performance comparison of different feature fusion methods.

Model	Acc(%)	F1 (%)	FPS	Params(M)
Simple concat	94.19	93.98	50.53	25.10
Swin Transformer	98.71	98.27	52.64	34.16

Ablation experiments have also indicated performance drops to a different degree when removing individual modalities or modules. Removing numerical data resulted in the largest drop in accuracy, which indicates that biomarkers are highly critical for disease classification. On the other hand, removing imaging reports resulted in a very small decrease in performance, although they were able to add supplementary value in some of the cases. These differences provide insights into future model optimization directions, suggesting that, in cases where data is limited, priority should be given to retaining the modalities that have the most significant impact on classification.

In summary, the ablation experiments provide strong evidence of the effectiveness of the proposed multimodal fusion framework and the design of its individual modules. Each modality and attention module plays a unique role in improving the accuracy and reliability of pneumonia classification. These results lay a solid foundation for the widespread application of multimodal deep learning models in real clinical practice.

### 4.4 Comparative experiments

To comprehensively validate the performance of the proposed PneumoFusion-Net multimodal deep learning framework in pneumonia classification tasks, a comparison was made with several classic unimodal deep learning models (Table 6).These models include ResNet50, VGG16, ResNet18, DenseNet, and Inception, which have been widely applied in medical image classification.

**TABLE 6:** Performance comparison of different models.

Model	Acc(%)	Params (M)	FLOPs(G)	FPS
ResNet50	87.35	23.52	4.13	187.20
VGG16	90.28	134.28	15.41	100.26
ResNet18	76.69	11.17	1.74	268.09
DenseNet	87.50	6.95	2.82	43.24
Inception	89.24	21.80	5.74	36.00
PneumoFusion-Net (ours)	98.96	34.16	8.67	59.35

**FIGURE 8:**
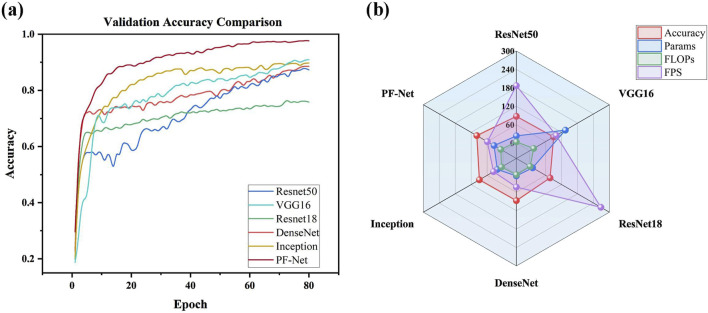
**(A)** Validation accuracy comparison of different models. **(B)** Radar Chart of performance metrics for model comparison (accuracy, parameters, FLOPs, FPS).

First, regarding classification accuracy, both PneumoFusion-Net and the Inception model achieved the highest accuracy of 98.96%, demonstrating the clear advantage of multimodal data fusion in improving classification accuracy. In contrast, ResNet50 and DenseNet achieved accuracy rates of 87.35% and 87.50%, respectively (Figure 8A). Although these models perform well in single-modality image analysis, they still exhibit limitations in multi-class pneumonia classification tasks. VGG16 outperformed ResNet50 and DenseNet with an accuracy of 90.28%, indicating that its deeper network structure has advantages in feature extraction. However, ResNet18 had the lowest accuracy at 76.69%, showing that its shallower network structure is insufficient for handling complex tasks.

In terms of model complexity, VGG16, due to its deep network structure, has the highest parameter count (134.28M) and FLOPs (15.41G), but its inference speed is the slowest at 100.26 frames per second (FPS) (Figure 8B). In comparison, ResNet50 and ResNet18 are relatively lightweight, with parameter counts of 23.52M and 11.17M, respectively, and FLOPs of 4.13G and 1.74G. Their inference speeds were 187.20 FPS and 268.09 FPS, respectively. DenseNet, with its efficient network design, has a parameter count of only 6.95M and FLOPs of 2.82G, but its inference speed is lower at 43.24 FPS, and it did not significantly outperform ResNet50 in terms of accuracy.

The Inception model achieves an excellent balance between high accuracy and computational efficiency. It has a parameter count of 21.80M, FLOPs of 5.74G, and an inference speed of 36.00 FPS, showing that it performs well in complex tasks with balanced performance. It has a bit higher parameters and FLOPs compared with Inception: 29.81M parameters and 8.67G FLOPs, respectively. On the contrary, a high speed of inference brings the best results for computational efficiency, ensuring great performance in multimodal fusion tasks as high as 59.35 FPS, and allows great potential for real-world applications.

In a nutshell, PneumoFusion-Net lays out excellent performance both from the perspective of classification accuracy and inference efficiency. Although its computational complexity is larger compared to some unimodal models, it fuses multi-modal information effectively and therefore enhances classification performance very much, especially when handling complex and atypical pneumonia cases. Results further strongly validate the potential of multimodal deep learning frameworks for clinical applications, especially in those diagnosis scenarios that need high accuracy and efficiency. PneumoFusion-Net demonstrates good application value in these contexts.

### 4.5 Attention visualization

**FIGURE 9:**
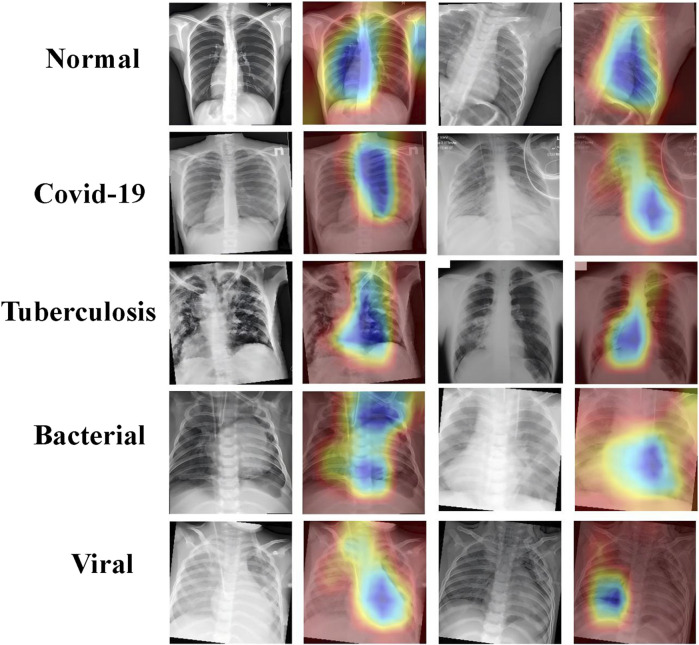
Grad-CAM visualizations highlighting key regions in chest X-rays for normal, COVID-19, tuberculosis, bacterial, and viral pneumonia.

Attention visualization was done on the CT images using Grad-CAM to understand the decision-making process of the PneumoFusion-Net model in multimodal fusion. Grad-CAM is a technique for interpreting deep learning models, highlighting an important region of an image for the decisions taken by the model. The heatmaps illustrate the region the model has focused on while classifying different types of pneumonia (Figure 9).

In the case of normal lungs, the attention heatmap indicates that there is an even distribution of attention throughout the lung fields without any outstanding highlighted region. The whole structure and the averageness of the tissue distribution make the model observe a healthy lung.

That is, in COVID-19 cases, model attention focuses around the peripheral lung area where GGOs usually occur. Such areas clearly show saliency in the heatmaps and confirm that GGO is one of the relevant features for COVID-19 diagnosis.

For tuberculosis, this model tends to give much attention to the upper lobes, which aligns with clinical observations that the upper parts of the lungs are more likely to be affected by tuberculosis. As can be observed from this attention visualization, the model effectively captures this critical feature within the diagnosis of TB.

The heatmaps of bacterial pneumonia cases reflect how the model mostly focuses on the center part of the lungs, which is usually where consolidation is found. This attention pattern underlines quite well the performance of the model in correctly identifying bacterial pneumonia by focusing its attention on areas of dense lung opacities.

In viral pneumonia, the attention of the model seems to be more dispersed, while there is attention to the lower lobes of the lungs. Heatmaps emphasize the presence of areas of ground-glass opacities, therefore pointing out how the model differentiates viral pneumonia, paying more attention to these characteristic features.

These attention visualization results further demonstrate the transparency of the decision-making process of PneumoFusion-Net and validate that the model effectively identifies key radiological features associated with the different types of pneumonia. In this way, the present model enhances not only the interpretability of the model but also represents a more reliable tool for clinical usage. Radiologists and clinicians can use such visual insights to understand the rationale behind the model prediction and provide valuable support for their clinical diagnosis.

## 5 Discussion

The proposed multimodal deep learning framework, namely PneumoFusion-Net, classifies cases of pneumonia with very good performance, outperforming several other unimodal and classic deep learning models. Normally, the diagnosis of pneumonia is based on a variety of data, such as imaging, clinical text, and laboratory tests. By incorporating the additional information of CT images, clinical text, numerical data, and imaging reports through effective fusion, PneumoFusion-Net managed to achieve the highest value of accuracy in classification up to 98.96%, hence giving it great potential for improving diagnosis precision by a great amount in complex medical tasks.

Unlike the unimodal approaches, the method leverages the complementary strengths of CT images, clinical text, and numerical data, each used in isolation for most of the works that composed the literature. The CT images will give a detailed insight into the anatomical and pathological changes within the lungs, while the clinical text and laboratory results offer essential contextual information of patient history and biomarkers, such as an elevated white blood cell count in bacterial infections. This cross-modal validation enables PneumoFusion-Net to make more robust and precise predictions, even when imaging features are ambiguous or atypical.

### 5.1 Model architecture and innovations

Therein, the architectural innovations of PneumoFusion-Net focus on an image processing module, text and numerical data processing modules, and a feature fusion mechanism that helps attain high overall model performance.

First, the novelty of the proposal is great within the well-known image-processing module: a Global Channel-Spatial Attention module, GCSA, and Depthwise Separable Convolution. GCSA allows one to capture significant features with coarseness in CT images by combining channel attention and spatial attention effectively. This proves particularly important for early pneumonia or when atypical imaging presentations, as ground-glass opacities or diffuse patterns are faint (Kim et al., 2019). Focusing on the image regions that are relevant for diagnosis, GCSA not only improved overall classification accuracy by 2.16%, but also enhanced model interpretability. Attention maps generated from this module provide clinicians with insight into how it decided this or that and thus give important support for early and differential pneumonia diagnosis.

As will be discussed below, Depthwise Separable Convolution embodies reduced computational complexity while maintaining performance for high-resolution medical images. In contrast to traditional convolution layers, depthwise separable convolution may reduce the number of parameters substantially by decoupling the processes of spatial and channel-wise filtering. With the reduction of computationally intensive processing, this model can process large-scale CT data with much higher efficiency while retaining high accuracy, thus solving one important challenge in medical image analysis called efficiency-performance balancing.

Then, the text processing module incorporates a natural language processing approach to extract semantic meaning from clinical text mainly through the use of pre-trained language models like BERT (Rogers et al., 2020). Clinical text data contains crucial information on patient history, symptoms, and other key clinical aspects related to pneumonia diagnosis. With the deep contextual understanding brought about by BERT, PneumoFusion-Net is able to represent unstructured natural language data in a meaningful way, enhancing the accuracy and robustness of the model in making the diagnosis of complex test cases where conditions could also be mutually inclusive.

Laboratory results also include WBC, NEUT%, and LYMP%, which are handled by a numerical data processing module. These numerical features are then processed through fully connected layers for informative pattern extraction after standardization and normalization. Biomarkers provide key diagnostic clues, especially in identifying bacterial and viral pneumonia. Experimental results also indicate that the inclusions of the numerical data raised the classification accuracy by more than 10% in distinguishing between types of pneumonia.

The architectural design of PneumoFusion-Net closely aligns with clinical diagnostic logic: radiologists typically first observe local lesions in CT (corresponding to the GCSA module), then combine laboratory indicators (residual MLP) and medical history text (BiLSTM-Attention) for comprehensive judgment. In contrast, existing methods [e.g., (Liang et al., 2020), (Li et al., 2021)] are structurally rigid and cannot simulate this dynamic process. For example, when CT findings are atypical, our model can reinforce the prediction weight of viral pneumonia through semantic cues like ‘elevated lymphocytes’ in the text, whereas traditional models rely solely on single-modality confidence.

### 5.2 Feature fusion mechanism

In this paper, the most significant novelty is represented by the mechanism of feature fusion, effectively integrating data that comes from different sources: a self-attention-based approach as a strategy to project features mapped from CT images, clinical text, and numerical data in a unified high-dimensional space. Such a feature fusion module captures relationships between different modalities and selects the most diagnostically relevant one through the self-attention mechanism. This way, it achieves excellence in the classification task where the image data alone is insufficient to diagnose completely. The complementary knowledge coming from the clinical text and numerical data provides an overall comprehensive and exact diagnosis.

This mechanism of fusion further enables the PneumoFusion-Net model to exploit the strengths of each modality to its fullest. CT images capture anatomy and pathologic features, while clinical text feeds rich semantic context, and numerical data provides quantitative evidence such as inflammatory markers. Such combinations yield a great improvement in classification performance. For instance, in distinguishing between bacterial and viral pneumonias, this kind of multimodal fusion approach employed by PneumoFusion-Net yielded a much better classification accuracy than was possible with unimodal models.

### 5.3 Ablation and comparative experiments

Further ablation experiments were conducted to illustrate more the contribution of each module involved: we systematically removed each modality and observed a steep drop in classification performance, underpinning the fact that multimodal integration is crucial. The removal of numerical data, for example, led to more than a 10% reduction in accuracy for the discrimination between bacterial and viral pneumonia, underlining the importance of laboratory data for the identification of types of pneumonia.

Comparatively, PneumoFusion-Net outperformed several unimodal and conventional deep learning models regarding both classification accuracy and inference efficiency. Admittedly, because multiple modalities and advanced attention mechanisms are involved, the number of parameters in the model is relatively high. This complexity is well-deserved given the significant improvement in performance. Most importantly, PneumoFusion-Net can be very effective for classifying complex cases, such as distinguishing viral from bacterial pneumonia, and was substantially superior compared to single-modality models.

### 5.4 Limitations and future directions

In spite of the remarkable achievements reported in this work, several areas require further attention and improvement to enhance the applicability and robustness of PneumoFusion-Net in clinical practice.

Although public dataset was utilized for training and validation, the diversity and scale may still be limited for broader clinical applications. Future research should incorporate datasets from various healthcare systems, countries, and imaging protocols. For instance, including data from underrepresented regions can account for variations in disease presentation and healthcare practices. Additionally, integrating CT images from different scanners and protocols, such as varying resolution or contrast settings, would improve the model’s adaptability across institutions. Domain adaptation techniques, such as adversarial learning, could also be employed to reduce performance degradation when applying the model to unseen datasets. These steps will improve the model’s robustness and ensure consistent performance across diverse clinical settings, enabling its relevance for global applications.

The current model treats data as static snapshots, whereas many clinical indicators, such as laboratory test results and symptom progression, evolve over time. Future research could introduce temporal modeling techniques to leverage such longitudinal data. Recurrent Neural Networks or Long Short-Term Memory networks could capture trends in laboratory values and temporal variations in imaging data, while Transformer-based temporal attention mechanisms might prioritize critical time points and assess disease progression patterns. For example, a temporal approach could identify worsening pneumonia cases by tracking rising inflammatory markers or emerging radiological changes over consecutive CT scans. Incorporating temporal dynamics would provide richer insights into disease trajectory, treatment responses, and long-term prognosis, enhancing the model’s diagnostic and predictive capabilities.

Although Depthwise Separable Convolution has reduced computational overhead, multimodal fusion frameworks remain resource-intensive. Further optimization strategies, such as pruning, quantization, or knowledge distillation, could create lightweight versions of the model without compromising performance. Neural Architecture Search could be explored to automatically design more efficient multimodal fusion architectures that balance performance and computational cost. Hardware acceleration, using GPUs, TPUs, or edge devices, could reduce inference time. For example, deploying a low-complexity version of PneumoFusion-Net on edge devices in rural clinics would enable real-time diagnosis with minimal infrastructure. These advancements would make the model scalable for time-sensitive and resource-constrained environments, enhancing its feasibility for real-world deployments.

Moreover, as large-scale pre-trained models (often referred to as “foundation models”) have shown significant potential in both vision and language domains, future research could explore integrating PneumoFusion-Net with these large models. For instance, Vision Transformers or large multimodal pretrained models could provide strong initial feature representations, reducing the need for extensive task-specific training data. Combining PneumoFusion-Net’s multimodal fusion strategy with large pre-trained models may yield even more powerful and generalizable feature extractors, thus further improving performance, particularly in low-data regimes or novel clinical scenarios.

To gain trust from clinicians, future iterations of the model should focus on improving interpretability. Advanced attention visualization methods, such as Shapley Additive Explanations (SHAP), could quantify the contribution of each modality to the final diagnosis. Additionally, developing user-friendly interfaces that allow clinicians to interact with model outputs, such as modifying or validating predictions based on additional patient data, would bridge the gap between AI predictions and clinical workflows. This would increase adoption and reliability in clinical practice.

In summary, future research should prioritize expanding datasets to improve diversity and generalization, integrating temporal dynamics to leverage longitudinal data, optimizing computational efficiency for real-world applications, exploring the integration of large-scale pre-trained models for robust and data-efficient feature extraction, and enhancing interpretability to align with clinical needs. By addressing these directions, PneumoFusion-Net and similar multimodal frameworks can evolve into robust, efficient, and trusted tools for clinical decision-making, significantly advancing healthcare outcomes and operational efficiency.

## 6 Conclusion

This study introduces PneumoFusion-Net, a multimodal deep learning framework that integrates CT images, clinical text, numerical data, and imaging reports, achieving superior classification performance. Experimental results demonstrate that PneumoFusion-Net achieves an accuracy of 98.96% and an F1 score of 98%, outperforming state-of-the-art models like ResNet50 and Inception by significant margins. Compared to a unimodal CT baseline, the model delivers a 14% absolute improvement in accuracy, highlighting the value of complementary cross-modal features. Additionally, leveraging Swin Transformer for feature fusion achieves a 4.52% accuracy gain over simple concatenation methods, underscoring the importance of advanced fusion strategies. These quantitative results confirm PneumoFusion-Net’s effectiveness in harnessing diverse data modalities for robust and accurate pneumonia diagnosis.The model excels in challenging pneumonia cases, such as distinguishing viral from bacterial infections, and attention-based visualizations enhance interpretability, offering clinicians insights into critical diagnostic factors. Each modality’s incremental contribution underscores the value of the proposed fusion strategy and the integrative attention design. Looking forward, future efforts will focus on incorporating diverse, international datasets for improved generalization; modeling temporal dynamics to capture evolving clinical indicators; exploring large-scale pre-trained models for more robust, data-efficient representations; and optimizing computational efficiency via pruning, quantization, and hardware acceleration. By addressing these directions, PneumoFusion-Net is poised to deliver more robust, scalable, and clinically meaningful AI-driven diagnostics, ultimately improving patient outcomes and accelerating the integration of AI in healthcare.

## Data Availability

The original contributions presented in the study are included in the article/supplementary material, further inquiries can be directed to the corresponding author.
